# Function, Adjustment, Quality of Life and Symptoms (FAQS) in Allogeneic Hematopoietic Stem Cell Transplantation (HSCT) Survivors: A Study Protocol

**DOI:** 10.1186/1477-7525-9-24

**Published:** 2011-04-17

**Authors:** Margaret F Bevans, Sandra A Mitchell, A John Barrett, Michael Bishop, Richard Childs, Daniel Fowler, Michael Krumlauf, Patricia Prince, Nonniekaye Shelburne, Leslie Wehrlen

**Affiliations:** 1 National Institutes of Health Clinical Center, 10 Center Drive, Bethesda, MD, USA; 2 National Institutes of Health, National Cancer Institute, 6130 Executive Blvd, Bethesda, MD, USA; 3 National Institutes of Health, National Heart, Lung, and Blood Institute, 10 Center Drive, Bethesda, MD, USA; 4 National Institutes of Health, National Cancer Institute, 10 Center Drive, Bethesda, MD, USA

## Abstract

**Background:**

The population of survivors following allogeneic HSCT continues to increase, and yet their experiences of recovery and long-term survivorship have not been fully characterized. This paper presents a study protocol examining over time the functional status, psychosocial adjustment, health-related quality of life, and symptom experience of survivors who have undergone allogeneic transplantation. The aims of the study are to: 1) explore the patterns of change in these health outcomes during the survivorship phase; 2) characterize subgroups of survivors experiencing adverse outcomes; and 3) examine relationships among outcomes and demographic and clinical factors (such as age, graft-versus-host disease (GVHD), and disease relapse).

**Methods:**

In this longitudinal observational study, adults who survive a minimum of 3 years from date of allogeneic transplantation complete a series of questionnaires annually. Demographic and clinical data are collected along with a series of patient-reported outcome measures, specifically: 1) Medical Outcomes Study SF- 36; 2) Functional Assessment of Chronic Illness Therapy (FACIT) - General, 3) FACIT-Fatigue; 4) FACIT- Spiritual; 5) Psychosocial Adjustment to Illness Scale; 6) Rotterdam Symptom Checklist-Revised; and 7) Pittsburgh Sleep Quality Index.

**Conclusions:**

This study will provide multidimensional patient-reported outcomes data to expand the understanding of the survivorship experience across the trajectory of allogeneic transplantation recovery. There are a number of inherent challenges in recruiting and retaining a diverse and representative sample of long-term transplant survivors. Study results will contribute to an understanding of outcomes experienced by transplant survivors, including those with chronic GVHD, malignant disease relapse, and other late effects following allogeneic transplantation.

**Trial Registration:**

ClinicalTrials.gov: NCT00128960

## Introduction

Allogeneic hematopoietic stem cell transplantation (HSCT) is an established and potentially curative treatment for various hematologic diseases [1-3]. More than 50 years after the first reports of bone marrow grafting, the National Marrow Donor Program (NMDP) reports approximately 20,000 allogeneic transplants in the U.S. annually [4]. Allogeneic HSCT has become standard therapy for patients with a wide variety of indications [5]; coupled with the increased availability of unrelated donors, and the use of cord blood as a stem cell source [6], the number of transplant recipients continues to grow.

The toxicity profile associated with an allogeneic HSCT is prominent. Significant toxicities result from the intense chemotherapy and radiotherapy utilized to prepare recipients, and from acute and chronic graft-versus-host disease (GVHD) that results from donor anti-host immune response against normal host tissues. Despite the toxicities, most 2-year survivors are cured of their original disease, yet their mortality rate remains higher than that of an age-matched healthy population [7]. The 3-year mortality following allogeneic HSCT ranges from 30-60%, depending upon factors such as age, co-morbid illness, malignant disease status at the time of transplantation, intensity of the preparative regimen, source of stem cell graft, and the extent of histo-incompatibility between donor and recipient [8,9].

Although there are an estimated 150,000 individuals living in the US who have survived 5 years or more following allogeneic HSCT [6], the patterns of recovery relative to functional status, psychosocial adjustment, quality of life and symptom distress in survivors 3 or more years following transplant are not fully understood. There have been several recent systematic reviews [10,11], but our knowledge of the recovery process is incomplete. For example, survivors of autologous HSCT are included in many of the study samples [11]; however, these observations may not generalize to survivors of allogeneic HSCT. Whereas the early recovery period following autologous and allogeneic HSCT may be somewhat comparable, allogeneic HSCT survivors encounter unique complications, such as acute and chronic GVHD and opportunistic infections related to the need for prolonged courses of immunosuppression. These conditions can be anticipated to substantially shape the symptom experience, functional status, psychosocial health, and health-related quality of life (HRQL) experienced by long-term survivors of allogeneic HSCT. Previously studied samples have also tended to represent a limited range of cultural and ethnic diversity, and there has also been a trend for HSCT survivor studies to utilize cross-sectional rather than longitudinal designs.

The experience of subpopulations of transplant survivors, such as those receiving reduced intensity conditioning (RIC) regimens, has not been fully characterized. The availability of RIC regimens has extended the eligibility for allogeneic HSCT to patients with solid tumors, those who are older than 55 years of age, and/or those with significant co-morbidities. Importantly, long-term quality of life outcomes in survivors of such RIC regimens have had only limited evaluation [12,13].

Improved knowledge of the pattern and correlates of recovery and long-term survivorship can be applied to inform screening/surveillance in transplant survivors, and would guide the development and testing of supportive care strategies to improve clinical outcomes in this patient population. This paper presents the rationale for and design of a longitudinal study to examine functional status, adjustment, quality of life, and symptoms in a diverse sample of English and Spanish speaking adult patients who have survived 3 years or more following an allogeneic HSCT, some of whom are experiencing late effects of transplantation, including chronic GVHD and disease relapse.

## Background

The Centers for Disease Control (CDC) defines cancer survivors as individuals "who have been diagnosed with cancer and the people in their lives who are affected by the diagnosis, including family members, friends, and caregivers" [14]. The evidence base examining outcomes in cancer survivors continues to grow; however, allogeneic HSCT survivors are under-represented in these studies and have some unique needs due to the intensity of the treatment, requirement for prolonged immunosuppression, and their risk for a range of late health effects. Individuals who receive allogeneic HSCT require close surveillance for a wide range of complications and health effects that may be anticipated to occur during the early (HSCT day to day 100), mid- (day 100 to 1 year), and long-term (beyond 1 year) phases of recovery [15,16]. Despite reports of negative physical and psychosocial sequelae and poor health-related quality of life (HRQL) early after HSCT [17,18], several studies suggest that by the fifth year of recovery, the majority of long-term allogeneic HSCT survivors report good to excellent HRQL relative to healthy populations and relative to patients with other chronic diseases [19-22]. However, there is variability in the pattern of recovery, and some survivors experience persistent and late effects of the conditioning regimen and transplantation procedure.

Research exploring the functional status of patients who have undergone an HSCT suggests considerable variability in the pace and extent of functional recovery. Although most patients experience some level of functional impairment in the period immediately following the transplant, the majority report gradual improvement over time with functional status comparable, in 1 or more dimensions, to age-matched healthy individuals [23-27]. Some studies have documented that 5-year survivors return to a level of physical function comparable to their pre-transplant baseline [28] or to a level of physical function on par with that observed in survivors who received conventional dose chemotherapy only [29]. On the other hand, investigators have described functional impairments in 10-year survivors relative to population norms [25,29,30]. Residual difficulties reported by survivors include impairments in physical and cognitive function [24,31-33], as well as restricted role and occupational functioning [24,27,33,34].

Few studies have explored functional status longitudinally following allogeneic HSCT. For example, a small sample of 16 participants reported little change in functional status beyond a mean of 28 months post transplant, thereby suggesting that a recovery plateau in terms of functional status may be achieved at approximately 2 years post-HSCT [33]. Similarly, most improvements in physical functioning occurred between 90 days and 2 years post transplant, with complete recovery for about 75% of patients by 2 years; nonetheless, an additional 10-20% of patients made some improvement in functioning between 2 and 4 years post-transplant [35]. In contrast, particularly in patients older than 25 at the time of HSCT, recovery may be non-linear, with functional status continuing to improve significantly with time, suggesting that relative to younger survivors, the trajectory of recovery in older patients may be somewhat delayed [17].

A major factor associated with impairments in functional status following HSCT is chronic GVHD. The incidence of chronic GVHD is estimated to range from 30% to 70% based in part on the degree of HLA mismatch between the HSCT donor and recipient [36]. Survivors experiencing chronic GVHD report impairments related to physical functioning, incomplete resumption of social function, and sexual dysfunction [17,37-41]. Chronic GVHD has also been associated with greater psychological and social dysfunction [25,28].

Psychological and social recovery lags behind physical recovery in allogeneic HSCT survivors [28]. When compared to controls or chemotherapy-only survivors [29], allogeneic HSCT survivors report impairments in social and emotional functioning at 5 [30] and 10 [29] years following transplantation. Research suggests that the experience of long-term survivorship after allogeneic HSCT, associated symptoms, and late effects can cause negative changes in self-concept [42] and mood disturbance [32,43] including depression [32,35,44], anxiety [35,45], and psychosocial distress [32,46]. The impact on social recovery includes diminished social relationships and social function [32,47,48]. The rate of return to work is quite variable, with reports ranging from 50-84% [23,25,28]. In the family unit, concern over family well-being has been documented as a source of stress for HSCT recipients [43], and there is evidence that transplant survivors and their partners experience poor dyadic adjustment [32]. Difficulties relative to sexual function (desire, arousal, and orgasm) are often reported as an issue by HSCT survivors [23,24,45,49-52], with survivors of HSCT reporting higher psychosexual dysfunction compared with healthy subjects [34].

There is little systematic information to characterize the symptom experience of allogeneic HSCT survivors. Survivors who were 5-10 years post-transplant reported more symptoms than healthy controls [30] and survivors from chemotherapy alone [29]. Studies suggest that discomforting physical symptoms such as fatigue [25,27,53-55], dyspnea [24], problems with sleep quality [27,45,56], taste changes [45], oral dryness [47], eye problems [25,45], skin dryness [27], joint stiffness [25,27], memory problems [25], cough [45], vaginal dryness and dyspareunia [17], which may or may not be directly attributable to chronic GVHD, are prevalent in transplant survivors across the survivorship continuum.

Little is known about how these symptoms evolve across time or the consequences of symptom burden for functional status and psychosocial health [25,27]. Research suggests that some improvements occur in the prevalence and severity of distressing symptoms across the first 5 years of recovery following allogeneic HSCT [43]. However, studies suggest that symptoms such as moderate-to-severe fatigue, skin dryness, pain, joint stiffness, eye symptoms, dyspnea, and problems with sleep remain prominent difficulties [23,25,27,29,30,38,45,52,56]. In correlational analyses, greater physical symptom distress was associated with higher levels of psychological distress and maladjustment [22,43,57,58], and inferior HRQL [34]. In a mixed sample of autologous and allogeneic HSCT survivors in which non-fatigued and severely fatigued survivors were compared, HSCT survivors with severe fatigue reported significantly more sleep disturbance on single-item measures of sleep quality or insomnia [55].

Despite the persistence of discomforting symptoms, problems with functional status and psychosocial well-being, survivors report that they would make the same choice again to undergo HSCT [20,27,59]. Indeed, there is evidence that with long-term survivorship may come salutary effects including spiritual growth, greater appreciation for life, and enhanced interpersonal relationships [60]. In support of this conclusion, it has been observed that survivors who were 3 or more years post-transplant reported higher scores in the domains of social functioning, mental health, and vitality when compared to normative scores. In addition, psychological, interpersonal, and spiritual growth has been reported when compared to an age- and gender-matched healthy comparison group [32]. Studies in allogeneic HSCT survivors also suggest that meaning in life, defined as the general sense that one's life has purpose and coherence, is positively related to several indicators of psychological adjustment [61] and improved functioning [45].

The recovery process following allogeneic HSCT appears to be nonlinear and unique for each individual. For example, qualitative data indicates that there may be transitions in the sense of meaning and coherence across the survivorship journey [62]. A period of grieving and life re-evaluation may be experienced during the third year post-HSCT, whereas through the fifth year post-HSCT and beyond, those survivors with residual physical problems report despair and concern about their futures [63]. Similarly a temporary decline in HRQL may occur at 10 years post-transplant, followed by a return to previously enjoyed levels. These observations suggest that the 10-year post-transplant anniversary may induce a transient increase in anxiety that reverses once the milestone is passed [23].

This study was designed to overcome many of the aforementioned limitations of the current literature and to address an important gap in understanding by offering a prospective, longitudinal, multidimensional examination of functional status, psychosocial adjustment, HRQL, and symptoms in a cohort of survivors 3 or more years following allogeneic HSCT. Such knowledge can be applied to strengthen clinician decision-making, and to allow patients and families to anticipate the process of recovery and optimize their self-management. This research will also provide valuable information needed for the development and testing of interventions for patients at high risk for poor health outcomes during the survivorship phase.

## Methods/Design

### Study Design

This is an ongoing prospective, longitudinal, observational study, in which adult patients who have survived a minimum of 3 years from date of allogeneic HSCT complete an annual survey using a variety of patient-reported outcome measures. Survivors are approached for participation within 60 days of their annual transplant follow-up, with subsequent yearly surveys linked to the date of their enrollment.

### Study Aims

A revised version of the Wilson and Cleary [64] model of health-related quality of life is applied to describe the relationships among conceptually distinct dimensions and make explicit the health concepts that are related to HRQL. The major health concepts evaluated in this study are obtained by patient self-report and include: physical and mental functioning, HRQL, psychosocial adjustment, and spiritual well-being. Aspects of the symptom experience evaluated in this study include physical and psychological symptom distress, fatigue, and sleep quality. The primary aim of the study is to explore the patterns of change in the major health outcomes during the survivorship phase. We hypothesize that functional status, psychosocial adjustment, cancer specific quality of life, or symptoms will vary over 3 sequential annual time points as a function of 1 or more clinical covariates in patients 3 or more years following allogeneic HSCT. Secondary aims of the study include 1) to characterize groups of survivors at risk for impairment; and 2) to explore relationships between a wide array of demographic, clinical factors, and health outcomes determined to be critical during survivorship phase.

### Sample Recruitment

Approval to conduct the study was obtained through the National Heart Lung and Blood Institute's intramural institutional review board. Participants are eligible to participate if they are: 1) ≥3 years post-first allogeneic HSCT; 2) ≥18 years of age; 3) able to read and speak English or Spanish; and 4) have a life expectancy of at least 6 months. Each potential subject is approached, and, if willing to participate, provides written informed consent.

The goal is to recruit survivors during their face-to-face outpatient follow-up with the transplant team. Although the majority of subjects return at annual intervals for in-person clinical follow-up, this number decreases as the time from allogeneic HSCT increases. When a subject does not return for an in-person clinical follow-up, contact is made to confirm the mailing address and evaluate their clinical status. Study materials are subsequently mailed with instructions and pre-paid materials for survey return.

### Study Procedures

Recruiting a diverse sample relative to race/ethnicity and language has implications for the selection of the survey instruments and for resource management including the budget and workload management among study team members. At the time of study initiation, we determined that enrolling Spanish-speaking subjects who were predominantly from Latin America and relatively unacculturated, was not only feasible but would make a substantive contribution to our understanding of the post-transplant survivorship experience. The contextual model of HRQL informed the selection of variables that describe the cultural and socio-ecological characteristics of our sample, and guided the development of hypotheses about the effects of these characteristics on functional status, the symptom experience, and HRQL in transplant survivors [65]. The contextual model of HRQL model extends the predominantly individual-centered HRQL paradigm to include contextual factors such as acculturation, social support, living arrangements, and country of residence which may be central to quality of life outcomes among ethnically and socio-economically diverse populations.

All measures selected for this study are available in both English and Spanish, although this is not true of all possible measures considered for the study. Beyond the selection of a measure, investigators may also incur additional licensing fees for measures in each language beyond English. Additional budget considerations include mailing fees for subjects who do not return at least annually for clinical follow-ups, and/or who reside outside of the United States. Multiple clinicians who are fluent in Spanish serve as associate investigators on the study, providing language concordance to facilitate the consent process, survey administration and the collection of clinical data when appropriate.

Although the focus of the research is on aggregated group data, individual subject surveys are reviewed in real-time for clinically meaningful responses that might require immediate assessment and possible intervention. To address the ethical concerns in sharing these data between the research and the clinical teams, the informed consent process encourages subjects to communicate directly with their clinical provider if they identify a concern during survey completion. In addition, the study team discusses directly with individual subjects any concerns that are identified through survey responses, and recommends further discussion with their clinical providers. Evidence suggests that HSCT clinicians may not fully appreciate the declines in well-being or psychosocial health experienced by transplant survivors [49]; therefore, study team members with expertise in particular areas (e.g. fatigue management, sleep quality, psychological adjustment) offered the primary care team referral suggestions, when appropriate.

### Measurement

*Physical and Mental Health *is measured using the Medical Outcomes Study (MOS) Short Form 36 (SF-36). The SF-36 is a 36-item self-report measure of physical and mental health [66]. The 36 items evaluate 8 factors including: physical functioning, physical role functioning, emotional role functioning, social functioning, bodily pain, mental health, vitality, and general health. In addition to the individual subscale scores, 2 component summary scores, physical (PCS) and mental (MCS) are computed through aggregation of the subscales. The SF-36 was translated into Spanish through the International Quality of Life Assessment Project. Strong evidence of internal consistency reliability and construct validity has also been documented in Spanish-speaking samples [67-70].

*Health-related quality of life (HRQL) *is measured with the Functional Assessment of Cancer Therapy - General Version 4 (FACT-G*)*. The FACT-G is a 27-item self-report disease-specific quality of life (QOL) questionnaire that is divided into 4 primary domains: physical, social/family, emotional well-being, and functional well-being. The FACT-G provides an overall QOL score and also a score for each subscale. The Spanish version of the FACT-G (version 4) has demonstrated construct validity and evidence of strong internal consistency reliability (α = 0.89) in various groups of Spanish speaking oncology patients [71-74].

*Psychosocial adjustment *is measured with the Psychosocial Adjustment to Illness Scale-Self Report (PAIS-SR). The PAIS-SR is a 46-item measure of psychosocial adjustment to illness in terms of 7 primary domains of adjustment: health care orientation, vocational environment, domestic environment, sexual relationships, extended family relationships, social environment, and psychological distress [75,76]. The PAIS-SR provides a PAIS Total Adjustment Score in addition to a score for each subscale. The PAIS-SR is also available in Spanish with evidence of internal consistency reliability (α = 0.72) in a mixed sample of Hispanic and non-Hispanic women with cancer [77].

*Physical and psychological symptoms *are assessed with the Rotterdam Symptom Checklist (RSCL). The RSCL is a 39-item self-report questionnaire measuring the quality of life in terms of 4 domains: physical symptoms distress, psychological distress, activity level, and overall global life quality in various types of cancer patients undergoing various treatments [78,79]. For this study, only the physical symptom distress and psychological distress are assessed. The physical symptoms distress scale consists of 23 items referring to different physical symptoms that are general (e.g. headaches) or cancer specific (e.g. gastrointestinal distress). The psychological distress scale consists of 7 items that reflect psychological symptoms that may be experienced by cancer patients. A Spanish version of the Rotterdam Symptom Checklist is also available and has demonstrated strong internal consistency reliability (α = 0.76 PSDS; α = 0.80 PDS) and construct validity in Spanish-speaking cancer patients [21].

*Fatigue and Spiritual Well-Being *are measured with domain-specific subscales of the Functional Assessment of Chronic Illness Therapy (FACIT). The FACIT-Fatigue scale is a 13-item self-report measure of cancer related fatigue [73]. The FACIT-Spiritual is a 12-item measure of spiritual well-being in patients with a chronic illness, including cancer [73,80]. The FACIT-Fatigue and the FACIT-Spiritual have been translated into Spanish with evidence of reliability in Spanish-speaking cancer patients [73,74,80].

*Sleep Quality *is measured with the Pittsburgh Sleep Quality Index (PSQI). The PSQI is an 18-item self-report measure of perceived sleep quality. The PSQI provides a global score as well as domain scores for subjective sleep quality, sleep latency, sleep duration, habitual sleep efficiency, sleep disturbances, use of sleeping medications, and daytime dysfunction [81]. Although evidence of validity and acceptable reliability have been reported for the English version [82], the psychometric properties for the Spanish language version of the PSQI have not been reported, although the PSQI has been used to evaluate sleep quality in Spanish-speaking women with gynecological and breast cancer [83].

Self-reported demographic variables include age, gender, race, ethnicity, marital status, number of children, current living arrangement, employment status, education level, health insurance status, primary language, acculturation [84], country of current residence, birth, and when diagnosed with transplant-related illness. Clinical variables relative to the transplant procedure and current clinical status are abstracted primarily from the clinical research record and include primary disease, intensity of conditioning regimen (i.e. reduced vs. myeloablative conditioning), date of transplant, degree of donor-recipient HLA match, donor stem cell source, current stage of disease, performance status, current anti-cancer treatment, history of acute [85] and chronic GVHD, current grade [86] and severity [87] of chronic GVHD, and the intensity of current immunosuppressive regimen.

### Data Analysis

The primary objective is to describe the patterns of change in functional status, psychosocial adjustment, symptoms, and cancer-specific HRQL following allogeneic HSCT in patients ≥3 years from allogeneic HSCT. Data from a recently completed study of HRQL following allogeneic HSCT was used to determine the detectable effect size relative to the primary research questions with a sample of size 80 and an alpha level of 0.05 [12]. In that study, no difference in fit between a model that assumed compound symmetry and one that assumed an unstructured covariance matrix was found. Thus, we assumed compound symmetry in estimating power. Estimated correlations across various measurement times (day 0 to day 30, day 30 to day 100, day 0 to day 100) were 0.25 for the FACT-G, 0.48 for the PCS dimension of the SF-36 and 0.52 for the MCS dimension of the SF-36; however, we realize that the correlations might lessen due to a reduction in error variance across the 3 years that subjects participate. Means on the PCS dimension of the SF-36 ranged from 35 to 40 with standard deviations ranging from 8 to 10 based on samples of 58 to 75 subjects. MCS means ranged from 44 to 49 with standard deviations from 9 to 10.5, again for samples of 58 to 75 subjects. Because these are normed scores, we can assume that the means and standard deviations will be in these ranges for the 3 years of data collection.

We examined the detectable effect size under several options, and made assumptions that: 1) means would be in the same range for the 3 time periods that we proposed to measure; 2) correlations across time would be no higher than we found in the earlier study and might, in fact, be lower; and 3) standard deviations would be in a comparable range to prior studies. The parameters to estimate the effect size were varied so that it could be detected under different scenarios assuming a 1-way repeated measures design. The level of significance was fixed at 0.05, sample size fixed at 80, and power fixed at 80%. On that basis, we are confident we will be able to detect a small effect size for our 3 primary scales. All calculations were completed using nQuery Advisor^® ^(Statistical Solutions Ltd, Boston, MA).

Descriptive statistics were used to describe the clinical and demographic characteristics of the subjects. For all analyses, significance was set at α=0.05 including post-hoc analyses which will be exploratory. In addition, time post-transplant was examined as a covariate in the analyses. If the time post transplant was found to be significant, a separate analysis was performed with stratification of the subjects into patient subgroups by year post-transplant (e.g. 3-5 years and 6 or more years).

The study involves repeated measurements over time; therefore, methods of longitudinal analysis, such as mixed-effects models will be used to evaluate change over time for the primary analyses. Covariates, including biologic, physiologic, and treatment-related factors, are anticipated to influence HRQL and will be incorporated into the cross-sectional and longitudinal analysis as appropriate. Prior research suggests that the conditioning regimen [22], age at transplantation [27], indication for transplant (non-malignant hematological conditions, such as aplastic anemia, versus malignant conditions) [50], type of transplant (matched sibling versus unrelated donor) [49] and the number of post-transplant complications experienced [17] all influence overall HRQL, as well as functional status and psychosocial health. Chronic GVHD is consistently a negative predictor of HRQL outcomes [27,35,88], and more intensive regimens of immunosuppression have been shown to contribute to adverse changes in functional performance in chronic GVHD [41].

Additional analysis is exploratory and hypotheses generating to examine the relationships among study variables and change across annual time points for individual variables. Methods of correlation and regression analysis will be used to evaluate the relationships between outcomes of interest and study variables. Because the study involves repeated measurements, methods of longitudinal analysis, such as the generalized estimating equations, mixed-effects models and growth mixture modeling will be used to evaluate change in health outcomes over time.

## Discussion

This longitudinal observational study will improve the understanding of variations in, and predictors of, the patient experience of allogeneic transplant survivorship, in particular, new understanding related to aspects of the patient experience that have been previously understudied, including fatigue, sleep quality, symptom distress, and psychosocial adjustment. Subjects are enrolled 3 or more years beyond the date of HSCT--a time when the survivor is beyond the greatest risk for disease- or treatment-related mortality and is likely to have fully reintegrated [89] into social roles that were relinquished during the acute treatment phase. As more attention is given to characterizing the effects of treatment on survivors and their families, this study will determine the prevalence and associations between relevant factors and define subsets of patients at highest risk for poor outcomes. These aims will be accomplished with a longitudinal design and in a sample comprised exclusively of survivors of allogeneic HSCT. The study will be further enriched by the inclusion of subgroups that have been understudied in prior stem cell transplant survivorship research, including Spanish speakers, and individuals undergoing reduced intensity transplantation or receiving treatment for disease relapse following transplantation. The knowledge gained from this study will refine an understanding of the patient experience of transplant survivors, generate testable hypotheses for future research, direct priorities in the development, and testing of supportive care interventions for survivors of allogeneic transplant.

## Competing interests

The authors declare that they have no competing interests.

## Authors' contributions

MB and SM designed the study and are accountable for data acquisition and preparation of the manuscript. MB is a Clinical Nurse Scientist with at the NIH Clinical Center, Department of Nursing Research and Translational Science. SM was a Clinical Nurse Scientist at the NIH Clinical Center, Department of Nursing Research and Translational Science, and currently is a Research Scientist, Outcomes Research Branch, NIH, National Cancer Institute. MK, PP, NS, LW are involved in study coordination and data collection. JB, MB, DF, RC contributed to subject recruitment. All authors read and approved the final manuscript.
